# The need for self-management in patients with Persistent Depressive Disorder (PDD) and their caregivers: A qualitative study using Grounded Theory

**DOI:** 10.1192/j.eurpsy.2023.1757

**Published:** 2023-07-19

**Authors:** E. Solis, I. V. Carlier, N. Kamminga, A. M. van Hemert

**Affiliations:** 1 Psychiatry, Leiden University Medical Center, Leiden; 2 Psychiatry and Medical Psychology, Maastricht University Medical Center, Maastricht, Netherlands

## Abstract

**Introduction:**

The Persistent Depression and Self-Management Study is a mixed-methods pragmatic randomized controlled trial that evaluated the “*Patient and Partner Education Program for All Chronic Illnesses*” (PPEP4All) in patients with persistent depressive disorder (PDD) compared to care as usual (CAU). PPEP4All is a brief, structured self-management program that focuses on functional recovery and involves the partner/caregiver in the program. The latter may improve patient outcomes and reduce caregiver psychosocial burden related to PDD.

**Objectives:**

In addition to evaluating the cost- and clinical-effectiveness of PPEP4All, we conducted a nested qualitative study to deepen our understanding of how patients with PDD and their caregivers cope with chronic depression. Additionally we identify areas in which they require care and learn how they could benefit from a self-management program like PPEP4All.

**Methods:**

In the nested qualitative study, 28 patients (16 from PPEP4All, 12 from CAU) and 9 partners/caregivers agreed to participate. The in-depth semi-structured interviews took place at participant’s home, the main research location, or over telephone. For each interview, we used a topic list, which was initially evaluated in a pilot study of patients with PDD. All interviews were audio recorded, with consent from the participant, and transcribed verbatim. Data were analyzed using Grounded Theory, with a constant comparative analysis method, using Atlas.ti version 9 software.

**Results:**

Qualitative data are currently being analyzed. We expect to identify important themes relevant to the patient’s and caregiver’s personal experience and learn how they use and implement self-management in their lives.

**Conclusions:**

PPEP4All may help patients with PDD and caregivers learn important self-management techniques to effectively cope with chronic depression and its consequences, and thus, it may help them meet their needs for care.

**Disclosure of Interest:**

None Declared

